# Mid-life cyclists preserve muscle mass and composition: a 3D MRI study

**DOI:** 10.1186/s12891-023-06283-3

**Published:** 2023-03-20

**Authors:** Martin A. Belzunce, Johann Henckel, Anna Di Laura, Laura M. Horga, Alister James Hart

**Affiliations:** 1 Royal National Orthopaedic Hospital, Stanmore, HA7 4LP UK; 2 Instituto de Ciencias Físicas (ICIFI-CONICET), Center for Complex Systems and Brain Sciences (CEMSC3), Escuela de Ciencia y Tecnología, Centro Universitario de Imágenes Médicas (CEUNIM), Universidad Nacional de Gral. San Martín, Campus Miguelete, 25 de Mayo y Francia, (1650), San Martín, Buenos Aires Argentina; 3 Consejo Nacional de Investigaciones Científicas y Tecnológicas (CONICET), Godoy Cruz 2290, (1425), Buenos Aires, Argentina; 4 Centro Universitario de Imágenes Médicas (CEUNIM), Universidad Nacional de Gral. San Martín, Campus Miguelete, 25 de Mayo 901, San Martín (1650), Buenos Aires, Argentina; 5 Institute of Mechanical Engineering, University College London, University College London, Stanmore, HA7 4LP UK; 6 Institute of Orthopaedics and Musculoskeletal Science, University College London, Stanmore, HA7 4LP UK

**Keywords:** Physical activity, Cyclists, Muscle health, Intramuscular fat, Sarcopenia, Gluteus maximus

## Abstract

Physical activity and a healthy lifestyle are crucial factors for delaying and reducing the effects of sarcopenia. Cycling has gained popularity in the last decades among midlife men. While the cardiovascular benefits of cycling and other endurance exercises have been extensively proved, the potential benefits of lifelong aerobic exercise on muscle health have not been adequately studied. Our aim was to quantify the benefits of cycling in terms of muscle health in middle-aged men, using magnetic resonance imaging. We ran a cross-sectional study involving two groups of middle-aged male adults (mean age 49 years, range 30–65) that underwent Dixon MRI of the pelvis. The groups consisted of 28 physically inactive (PI) and 28 trained recreational cyclists. The latter had cycled more than 7000 km in the last year and have been training for 15 years on average, while the PI volunteers have not practiced sports for an average of 27 years. We processed the Dixon MRI scans by labelling and computing the fat fraction (FF), volume and lean volume of gluteus maximus (GMAX) and gluteus medius (GMED); and measuring the volume of subcutaneous adipose tissue (SAT). We found that the cyclists group had lower FF levels, a measure of intramuscular fat infiltration, compared to the PI group for GMAX (PI median FF 21.6%, cyclists median FF 14.8%, p < 0.01) and GMED (PI median FF 16.0%, cyclists median FF 11.4%, p < 0.01). Cyclists had also larger GMAX and GMED muscles than the PI group (p < 0.01), after normalizing it by body mass. Muscle mass and fat infiltration were strongly correlated with SAT volume. These results suggest that cycling could help preserve muscle mass and composition in middle-aged men. Although more research is needed to support these results, this study adds new evidence to support public health efforts to promote cycling.

**Supplementary Information:**

The online version contains supplementary material available at 10.1186/s12891-023-06283-3.

## Introduction

Sarcopenia is the progressive loss of muscle mass and function progressively as part of the natural ageing process [1–3]. Low levels of physical activity have also been associated with increased levels of muscle fat infiltration and progressive muscle weakness [4–6]. Therefore, physical activity and a healthy lifestyle are crucial factors in delaying and reducing the effects of sarcopenia. Moreover, larger muscle mass in early life can help to preserve muscle function at a later stage of life [7].

For this reason, being physically active before and during the onset of sarcopenia can have an important impact on later life stages to protect individuals against the effects of ageing on muscle health [3]. Cycling has gained popularity in the last decade, being one of the main physical activities in middle-aged adults [8], who are taking up cycling not only due to its physical health benefits and low impact, but also because of its effects on mental well-being, as shown by Glackin and Beale [9]. The benefits of cycling in terms of cardiovascular health and fitness have been widely studied [10–17]. For example, commuter cycling has been associated with improvements in cardiovascular fitness, reduction of all-cause mortality, cancer risk, overweight, and obesity among middle-aged individuals [10]. However, the impact of long-term cycling on muscle health has not been thoroughly explored [18, 19].

Consequently, it is essential to determine if cycling can help prevent sarcopenia and to estimate its impact on muscle mass and composition, which are markers associated with strength and mobility [20–23]. These two important muscle health markers can be quantified by measuring muscle volume and intramuscular fat (IMF) content from magnetic resonance imaging (MRI) [24, 25].

In this work, we aimed to study the benefits of cycling in terms of muscle health by comparing muscle health markers of two middle-aged men groups with different lifestyles: a group that has adopted cycling as their main recreational physical activity and a group of physically inactive subjects. We obtained Dixon magnetic resonance images of the pelvic region of each subject and computed the IMF content, muscle mass, and lean muscle mass of the gluteus maximus (very involved in cycling) and gluteus medius (less involved in cycling).

## Methods

### Study design

This was a cross-sectional study involving two matched groups of middle-aged adults who underwent MRI. The first group consisted of trained male cyclists that had cycled more than 7000 km in the preceding year. The second group consisted of physically inactive (PI) men (defined as men doing less than 1 h of physical exercise per week) ready to start the UK NHS (National Health Service) Couch to 5 K (Cto5K) programme, a running plan for absolute beginners. The inclusion criteria for this group were less than 1 h of physical exercise per week and registration to start the Cto5K programme. Common inclusion criteria for both groups were the absence of injuries and other health problems, no contraindication to MRI, and 30–65 years of age.

We recruited a total of 56 subjects, 28 for the physically inactive group and 28 for the cyclists group, who met the inclusion criteria. The median cycling experience for the latter group was 12 years. Demographic data for each group are presented in Table 1.

The volunteers underwent MRI and filled out a structured questionnaire regarding their physical activity levels and lifestyle on the scanning day. The following validated questionnaires were used: General Practice Physical Activity Questionnaire (GPPAQ), Warwick-Edinburgh Mental Wellbeing Scales (WEMWBS) [26] for mental health, and Hip disability and Osteoarthritis Outcome Score (HOOS) [27] for hip health as we assess two hip muscles. In addition, cyclists were asked about their cycling experience.

All subjects provided written informed consent. The study was approved by the UCL Research Ethics Committee (REC) [Number 13,823 /001].

**Table 1:** Demographics of the two study groups. Mean ± SD values are reported

		Physically Inactive	Cyclists	p-value
Demographics	Subjects	N = 28	N = 28	
	Age [years]	49.3 ± 10.6	48.0 ± 9.0	
	Body Mass [kg]	94.6 ± 17.7	77.2 ± 7.7	
	Height [cm]	179.1 ± 6.5	180.8 ± 6.8	
	BMI [kg/m^2^]	29.4 ± 5.0	23.7 ± 2.5	
General Health Questionnaires	Physical Activity (GPAQ*)	I = 12, MI = 4, MA = 9, A = 5	A = 28	
	WEWBMS^†^	48.3 ± 8.6	52.7 ± 7.7	p = 0.03
	HOOS^§^ Pain	95.1 ± 7.9	97.7 ± 6.2	p = 0.06
	HOOS^§^ Function, Daily Living	95.6 ± 7.8	98.8 ± 4.7	p = 0.01
	HOOS^§^ Function, Sports	92.4 ± 11.7	97.6 ± 5.6	p = 0.10

* General Practice Physical Activity Questionnaire; ^†^ Warwick-Edinburgh Mental Wellbeing Scales; ^§^ Hip disability and Osteoarthritis Outcome Score

### MRI acquisition

All subjects underwent a standardized MRI protocol. The MR images were acquired on a 3T scanner (Siemens Magneton Vida, Erlangen, Germany) using a body coil. The scanning protocol consisted of axial PD TSE Dixon and axial T1-weighted images with a field of view (FOV) that covered from 2 cm below the lesser trochanter to the top of the L1 lumbar spine vertebra. The PD TSE Dixon sequence had the following parameters: slice thickness 2.6 mm, spacing between slices 2.6 mm, repetition time (TR) 5590 msec, echo time (TE) 51 msec, number of excitations 1, number of echoes 14, flip angle 150°. The voxel size was 0.55 × 0.55 × 2.6 mm3.

### Muscle health assessment with MRI

We used gluteus maximus (GMAX) and gluteus medius (GMED) muscles to evaluate general muscle health, as they are essential to maintain an active lifestyle and are involved in a wide range of physical activities. Furthermore, GMAX is highly involved during the hip extension phase of the pedalling cycle [28, 29] but not GMED. Hence, we compared the health of a muscle that is directly trained by cycling with a muscle not very relevant in this sport. For each muscle, we computed three MRI-based muscle health metrics: intramuscular fat (IMF) content, muscle mass and lean muscle mass following a similar process to what we have done in previous studies [6, 30].

**Fig. 1:**
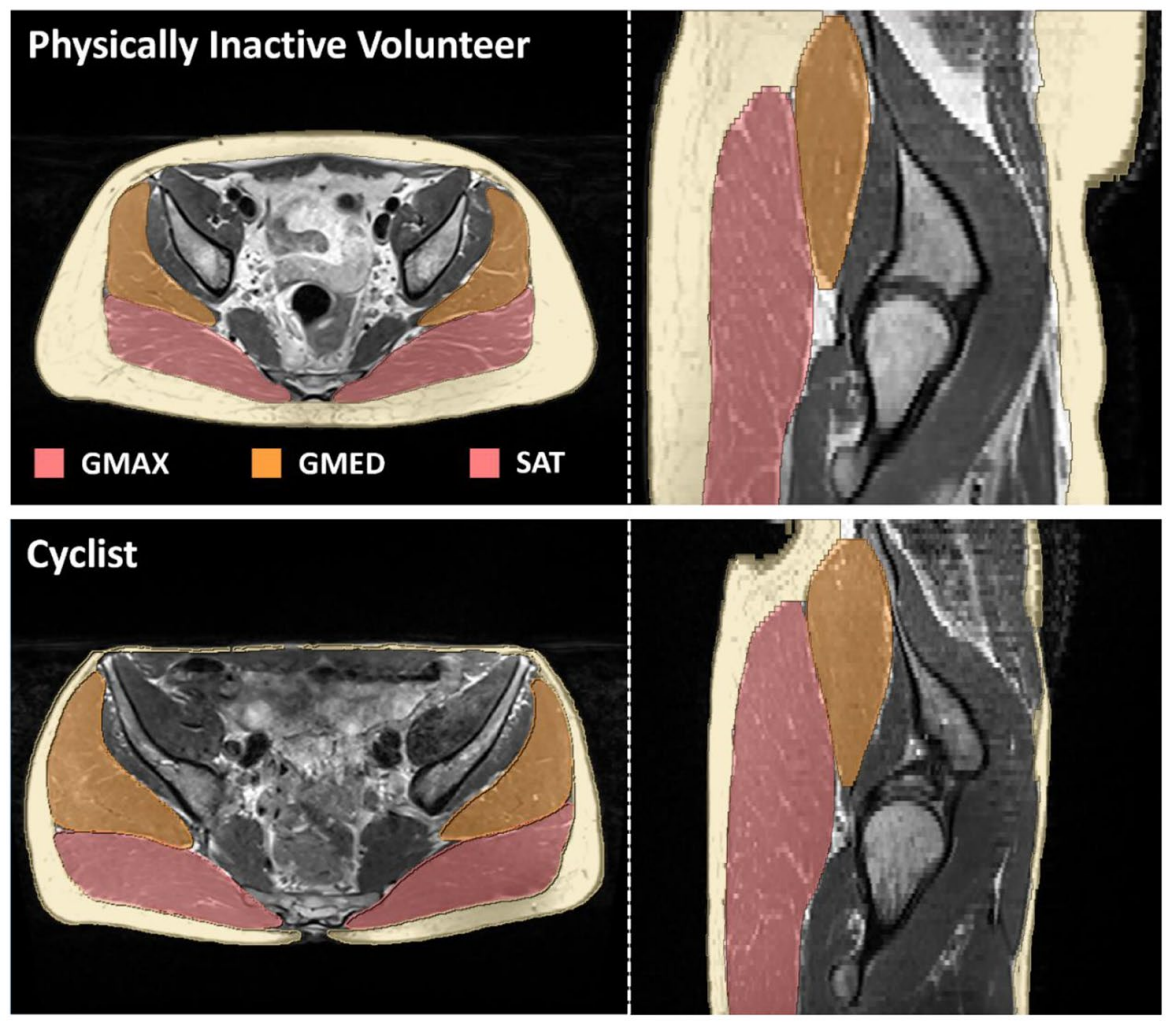
Axial and sagittal views of a physically inactive volunteer (top row) and a well-trained recreational cyclist (bottom row). The labels for GMAX, GMED and SAT are illustrated for both cases. The two subjects had GMAX fat fraction values of 21.8% and 17.6%

To measure the aforementioned metrics, we labelled the left and right GMAX and GMED muscles (see Fig. 1) using an in-house tool [25, 31] that runs on Simpleware ScanIP (Version 2021.3; Synopsys, Inc., Mountain View, USA). The tool has already been validated and used in other studies [6, 30]. The intramuscular fat (IMF) content was quantitatively measured by computing the mean fat fraction (FF) on each label from the FF Dixon MR images [32–34]. Muscle mass was estimated by summing up all voxels within a label and multiplying the results by the voxel size. Lean muscle mass was estimated as volume multiplied by (1-FF). Both volumetric measurements were normalized by the body mass of each subject. All the MRI scans were cropped at the tip of the lesser trochanter (LT) to avoid volume differences due to FOV mismatches.

Additionally, size profiles were computed from the cross-sectional areas (CSA) of each axial slice that forms a muscle label. CSAs were also normalized by body mass. Profiles for FF and lean CSA were also computed. All the profiles (with a different number of slices for each subject) were resampled into 50 fixed slices or sampling points by applying a linear interpolation as described in a previous work [30]. We computed the median and the IQR for each slice of the resampled CSAs profiles and then estimated the relative percentage difference between the two groups.

### Subcutaneous adipose tissue

We measured the amount of subcutaneous adipose tissue (SAT) in the pelvis region by labelling the SAT on the Dixon MRI images and computing its volume (V_SAT_) and normalized volume (NV_SAT_) by body mass. The labelling was performed with an automated algorithm that classifies each voxel into three different classes as proposed by Bezrukov et al. [35], and then subtracts a convex hull of the non-fat mask from the fat label for each slice.

### Statistical analyses

We computed nonparametric descriptive statistics for the FF and volume values for each muscle and group, since their distribution was not normally distributed (Kolmogorov-Smirnov test, p < 0.01). We compared the FF, volume and lean volume of the GMAX and GMED muscles, and the SAT volume, for the PI and cyclists groups using a Mann-Whitney U test for samples not normally distributed. Effect sizes were computed using the *r-*value, defined as $$\raisebox{1ex}{$Z$}\!\left/ \!\raisebox{-1ex}{$\sqrt{N}$}\right.$$, where *Z* is the standardized value for the U-value of the test [36]. Effect sizes were classified in low (*r* < 0.3), medium (0.3 < *r* < 0.5) and large (*r* > 0.5).

We performed a linear regression analysis between cycling (as a categorical variable) and FF and NV. In addition, a multiple regression analyses were used to adjust for potential covariates. The variables tested were BMI, age, weight, NV_SAT_, hip health (using three HOOS scores) and levels of physical activity as defined by the GPAQ.

We used a level of statistical significance (α) of 0.05 for all the tests.

## Results

The PI group had a larger body mass (median 92.5 kg; p < 0.01) and a higher BMI (median 28.5 kg/m^2^; p < 0.01) than the cyclists group (median body mass 76.0 kg, median BMI 23.7 kg/m^2^). In the PI group, 16 volunteers were classified as inactive, 9 as moderately active and 5 as active using the GPAQ scores. All participants reported good hip function and health as assessed with the HOOS questionnaire, where we only found differences between the PI and the cyclists groups for the scores “HOOS Function, Daily Living” (Table 1).

We found that the cyclists group had lower levels of fat infiltration for the two muscles under analysis compared to the PI group, and had larger GMAX and GMED muscles after normalizing the muscle volume by body mass. In Table 2, the median (IQR) values of fat fraction, volume, normalized volume and normalized lean volume are shown for each group, as the well as SAT volume and normalized volume.

**Table 2:** Median (IQR) values for GMAX and GMED muscles for the PI and Cyclists groups

	Physically Inactive	Cyclists	p-value
Fat Fraction [%]			
GMAX*	21.6 (19.4–25.0)	14.8 (13.3–16.2)	p < 0.01
GMED^†^	16.0 (14.8–17.1)	11.4 (10.5–12.8)	p < 0.01
Volume [cm3]			
GMAX	804.7 (696.8-914.4)	791.3 (707.6-869.1)	p = 0.79
GMED	414.5 (373.1-484.5)	390.2 (359.2-412.4)	p = 0.09
Normalized Volume [cm3/kg]			
GMAX	8.6 (8.0-9.2)	10.2 (9.5–11.0)	p < 0.01
GMED	4.5 (4.3–4.7)	5.0 (4.8–5.2)	p < 0.01
Lean Normalized Volume [cm3/kg]			
GMAX	6.5 (5.8–7.5)	8.6 (8.1–9.5)	p < 0.01
GMED	3.7 (3.5-4.0)	4.4 (4.2–4.6)	p < 0.01
SAT^§^ Volume [cm3]	5071 (3454–6642)	2158 (1858–2791)	p < 0.01
SAT Normalized Volume [cm3/kg]	53.2 (42.7–62.7)	29.3 (24.9–34.4)	p < 0.01

^*^ Gluteus Maximus; ^†^ Gluteus Medius; ^§^ Subcutaneous Adipose Tissue

### Intramuscular fat

**Fig. 2:**
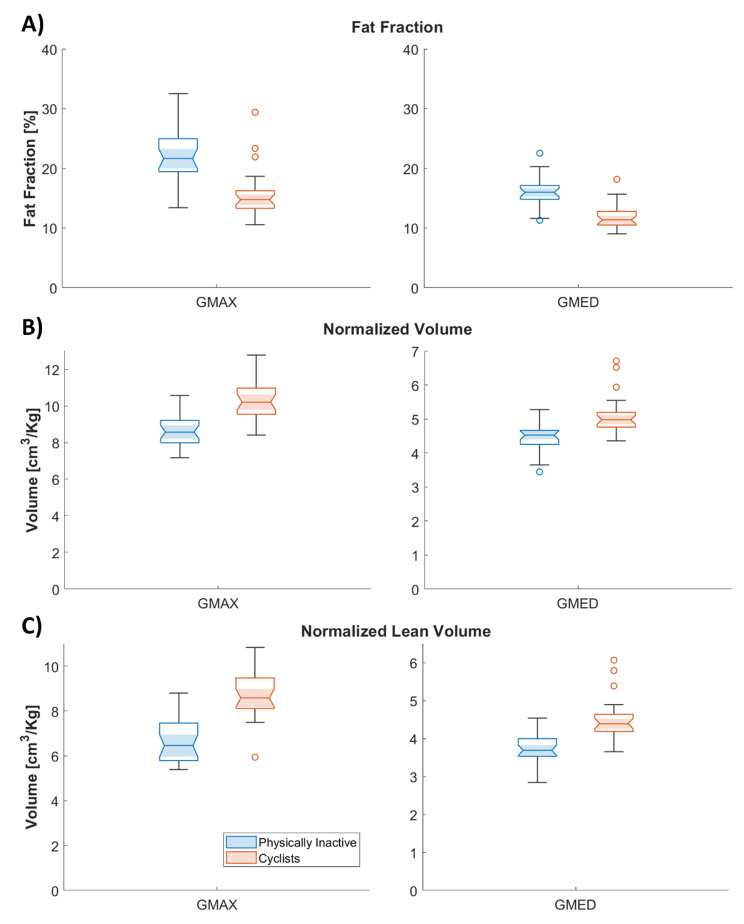
Boxplots of FF (A), normalized volume (B) and normalized lean volume (C) values of GMAX and GMED muscles for each group. On each box, the central mark is the median and the edges of the box are the 25th and 75th percentiles. Outliers are plotted individually with circles

In Fig. 2A, we show boxplots of FF for each group. The FF values were lower for cyclists for GMAX (p < 0.01, large effect size *r* = 0.61) and GMED (p < 0.01, large effect size *r* = 0.69). FF was correlated with BMI (R^2^ = 0.588, p < 0.01 for GMAX; R^2^ = 0.496, p < 0.01 for GMED), the categorical variable PI/Cyclists (R^2^ = 0.369, p < 0.01 for GMAX; R^2^ = 0.357, p < 0.01 for GMED) and NV_SAT_ (R^2^ = 0.607, p < 0.01 for GMAX; R^2^ = 0.582, p < 0.01 for GMED).

The multivariate model with highest prediction power included BMI and the PI/Cyclists variable as predictors. The NV_SAT_ was highly correlated with both predictor variables and was excluded from the analysis to avoid collinearity. Age, levels of physical activity (as measured with the GPAQ) and hip health were not predictors of FF.

The multivariate models for FF prediction were:

$$\begin{array}{l}F{F_{GMAX}}[\%] = - 1.3 + 0.7*BMI + 2.5*PI\,\\\,({R^2} = 0.629,\,{p_{BMI}} < 0.01,\,{p_{PI}} = 0.02)\end{array}$$

$$\begin{array}{l}F{F_{GMED}}[\%] = 4.7 + 0.3*BMI + 2.4*PI\,\\\,({R^2} = 0.601,\,{p_{BMI}} < 0.01,\,{p_{PI}} = 0.01)\end{array}$$

### Muscle mass

In Fig. 2B C, we show boxplots of NV and LNV (B) for each group. NVs were larger for the cyclists than for the PI group for both GMAX (p < 0.01, large effect size *r*=-0.72) and GMED (p < 0.01, large effect size *r*=-0.55). The same was observed for the LNV (GMAX, large effect size *r*=-0.7391; GMED, large effect size *r* = 0.6515).

Normalized muscle volume was correlated with the PI/Cyclists categorical variable (R^2^ = 0.439, p < 0.01 for GMAX; R^2^ = 0.294, p < 0.01 for GMED) and the NV_SAT_ (R^2^ = 0.607, p < 0.01 for GMAX; R^2^ = 0.582, p < 0.01 for GMED), and weakly correlated with BMI (R^2^ = 0.233, p < 0.01 for GMAX; R^2^ = 0.226, p < 0.01 for GMED).

The multivariate model with highest prediction power included the NV_SAT_ and PI/Cyclists variables as predictors of the GMAX normalized volume:

$$\begin{array}{l}N{V_{GMAX}} = 1148 - 32*NSAT\,[c{m^3}] - 1256*PI\,\\\,({R^2} = 0.629,\,{p_{NSAT}} = 0.04,\,{p_{PI}} < 0.01)\end{array}$$

The correlation coefficients between all the tested variables and between the main output variables (FF and NV) and the predictors are illustrated in Figure S.1 and Figure S. 2 of the Supplementary Material. An exploratory data analysis of these variables is shown in Figure S. 3, where the differences between the two groups under study can be easily seen.

### Intramuscular fat and CSA profiles

**Fig. 3:**
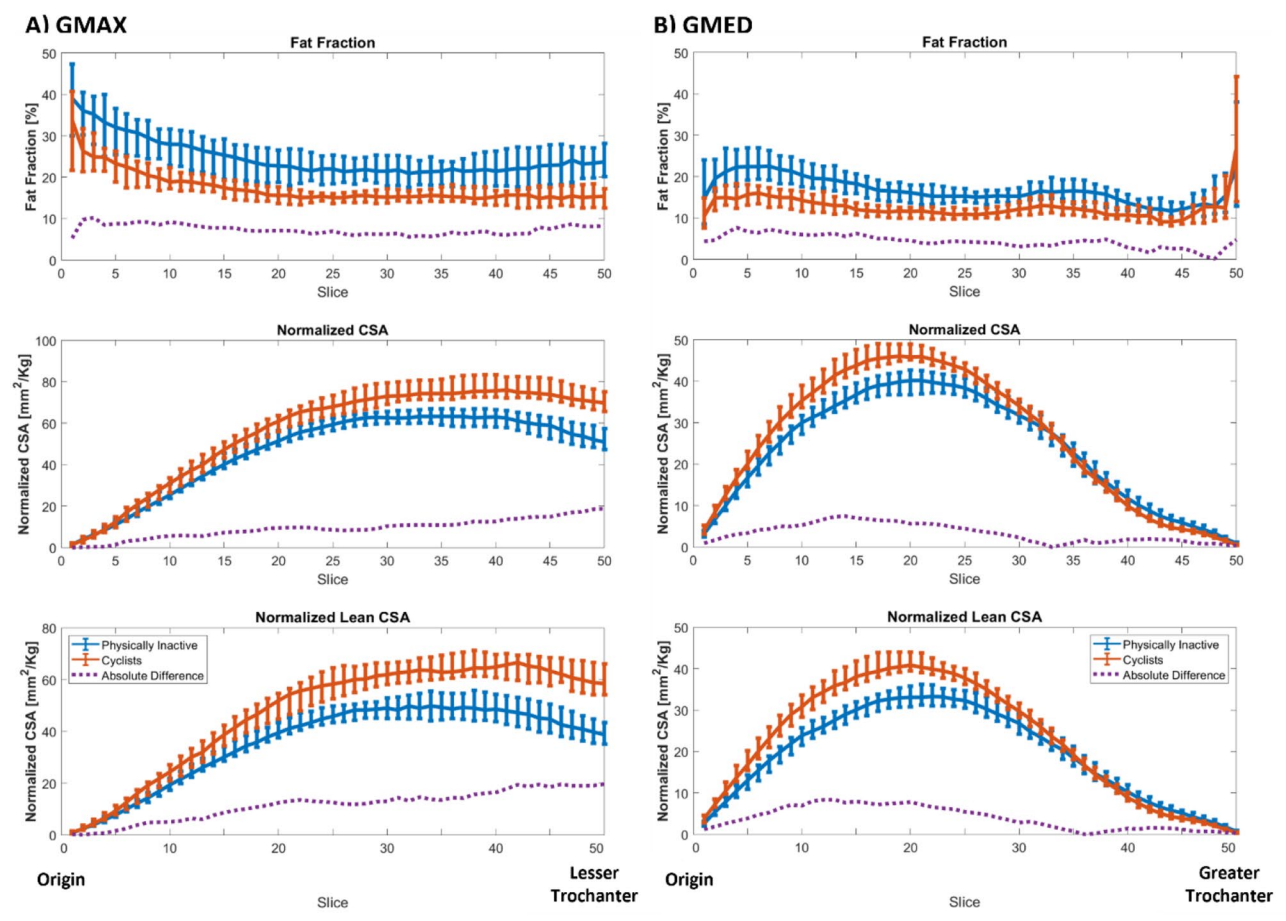
Axial profiles with median values and IQR error bars for GMAX fat fraction (A), normalized cross-sectional areas (B) and normalized lean cross-sectional areas (C) for the PI (blue) and cyclists (red) groups. In a purple dashed line and using the left y-axis, the relative percentage difference between the two groups is shown for each slice. The profiles go from the origin of GMAX (slice 1) to the level of the lesser trochanter (slice 50, the most inferior slice)

In Fig. 3, we show axial profiles of GMAX (A) and GMED (B) for the PI and Cyclists groups, where the median FF, CSA and lean CSA are shown for each axial slice. The error bars represent the IQR in each slice. The absolute percentage difference between the two groups is shown in a dashed line, where the higher FF of the PI group is uniform along both muscles. In terms of muscle size, the differences were more substantial in the inferior section near the lesser trochanter for GMAX and in the superior region for GMED.

## Discussion

This was a cross-sectional study in which two matched groups of middle-aged men were compared. Our objective was to quantify of cycling on muscle health in midlife men. We showed that the cyclists group had lower levels of intramuscular fat and greater muscle mass for both GMAX and GMED muscles than the physically inactive middle-aged men, with large effect sizes. These are relevant findings, as they suggest that cycling, an activity that is increasingly popular among middle-aged men [8], could be effective in slowing the degradation of muscle composition and the loss of muscle mass that is typically observed in the ageing population.

Although our results are somehow expected and that more research is needed to understand how much cycling is needed to observe these outcomes, we provide important evidence supporting that lifelong aerobic exercise can slow the loss of muscle mass and function. These are novel results as the research in the prevention of sarcopenia has been mainly focused on resistance training as intervention instead of aerobic exercise [19]. A previous study showed that the thigh muscle mass of highly trained master cyclists was comparable to healthy young adults [37], which agrees with our findings regarding muscle mass preservation. In addition, our quantitative metrics from Dixon MRI offer reference values that can be used to study other groups in the future (i.e. commuter cyclists instead of the highly/moderately trained midlife cyclists of our study).

We focused on only men due to the high cost of MRI scans, which allowed us to achieve a good sample size for two well-matched groups. Muscle health was assessed for GMAX, greatly involved in cycling, and GMED to determine if the benefits of cycling were only seen in the muscles targeted by this sport. We used Dixon FF as a quantitative measure of intramuscular fat, and muscle volume normalized by body mass as a measure of muscle mass.

IMF levels were associated with a larger volume of SAT in the pelvis area, a higher BMI, and not being in the cyclists group. The high IMF and pelvic SAT observed in the PI group could be potentially associated with metabolic impairment [38]. Despite the lower levels of IMF content of the Cyclists groups compared to the PI groups, the former had higher levels of fat infiltration compared to previously published reference data of the gluteal muscles in healthy active individuals [30, 39]. This could be explained by the fact that in our study the participants were considerably older. GMAX and GMED muscles had different FF range in agreement with previously reported values [30, 39].

The larger muscle mass of the cyclists is an expected effect of training as muscle volume is associated with strength and power [39–42]. Using CSA profiles, we found that the size difference was located mainly in the inferior section of GMAX, which could be explained by the fact that GMAX is heavily involved during the hip extension phase of the pedaling cycle [28]. The inferior section of GMAX is mainly active during hip extension, while the superior section of GMAX is mainly active for abduction and external rotation that are not relevant for cycling [40]. Although GMED is not particularly targeted during cycling, we found meaningful differences between the two groups in the superior region of the muscle, which could be explained by GMED being active when using a more posterior pedal position [28].

The combination of lower IMF and larger GMAX and GMED mass in the cyclists groups, translated in even larger differences for lean muscle mass (normalized by body mass), a measure that combines muscle size (defined as the volume within the muscle fascia) and composition. The effect sizes on lean muscle mass of not being a cyclist were large for both GMAX and GMED, although slightly higher for the former.

A limitation of this work is that we assessed the impact of cycling only in the gluteal muscles, which is an important muscle group associated with good mobility and a reduced risk of falls in the elder population [41, 42], but further research is needed to study if this muscle group is representative of the overall muscle health of middle-aged individuals. A second limitation of this study is that the two groups were recruited according to their current levels of physical activity. However, the volunteers of the cyclists group had been practicing this sports for a mean time of 12 years, and most of the PI subjects reported a lifelong physical inactivity. Therefore, this study compared two groups of midlife men with different long-standing lifestyles, although self-reported. A third limitation was that the recruiting criteria were based on self-reported physical inactivity, but half of the participants in the PI group were classified as moderately active or active using the GPAQ questionnaire.

## Conclusion

We observed that well-trained midlife recreational cyclists had lower levels of fat infiltration and greater muscle mass for the two main gluteal muscles when compared to physically inactive individuals of the same age. This suggests that, in addition to other previously reported benefits, cycling could help preserve muscle health in middle-aged men. Although more research is needed to know at what level and how many years of cycling are required to see its positive impact on muscle health, this study adds new evidence to support public health efforts to promote cycling.

## Electronic supplementary material

Below is the link to the electronic supplementary material.

Supplementary Material 1

## Data Availability

The data analysed during the current study is available from the corresponding author on reasonable request.
