# Supplementary material for: Mid-life cyclists preserve muscle mass and composition: a 3D MRI study
Source: BMC Musculoskelet Disord. 2023 Mar 20;24:209. doi: 10.1186/s12891-023-06283-3 (PMC10026522; doi:10.1186/s12891-023-06283-3)
Supplement: Supplementary file 1 — Supplementary Material 1 [file 12891_2023_6283_MOESM1_ESM.pdf]

## Supplementary Material

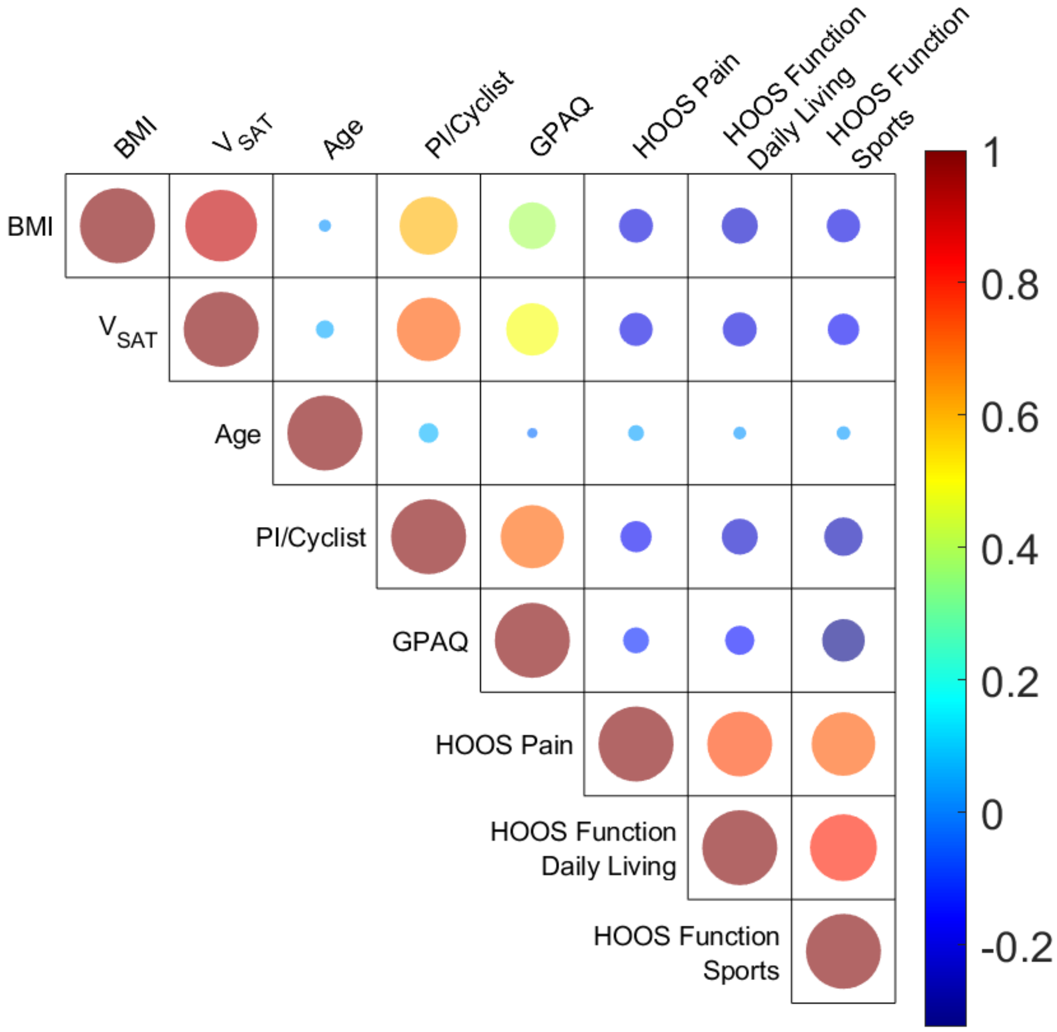


Figure S. 1. Plot of the correlation coefficients between the covariates tested as predictors of FF and NV. The size and colour of each circle represents the level and sign of the correlations between variables.

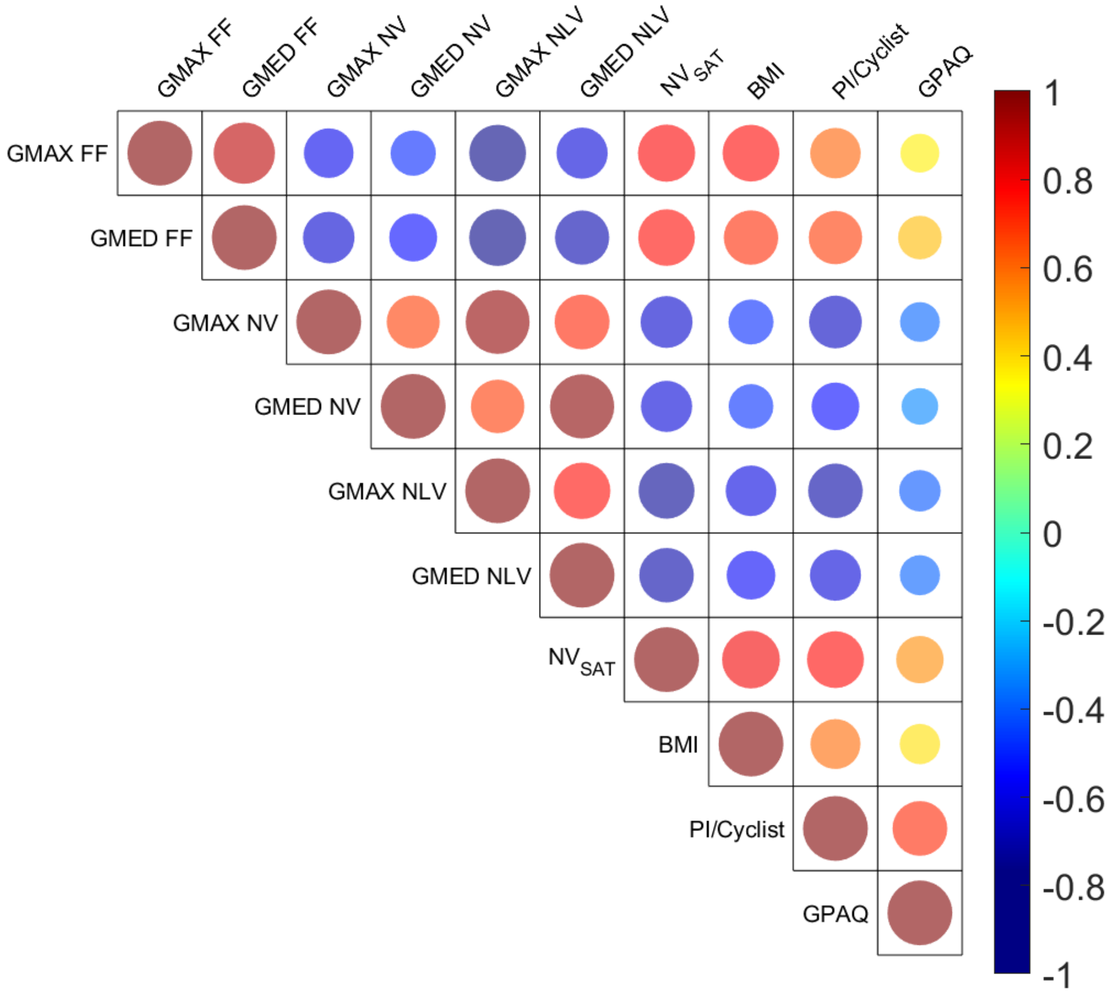


*Figure S. 2.* Correlation coefficients between the main variables involved in muscle health for the physically inactive and cyclists groups.

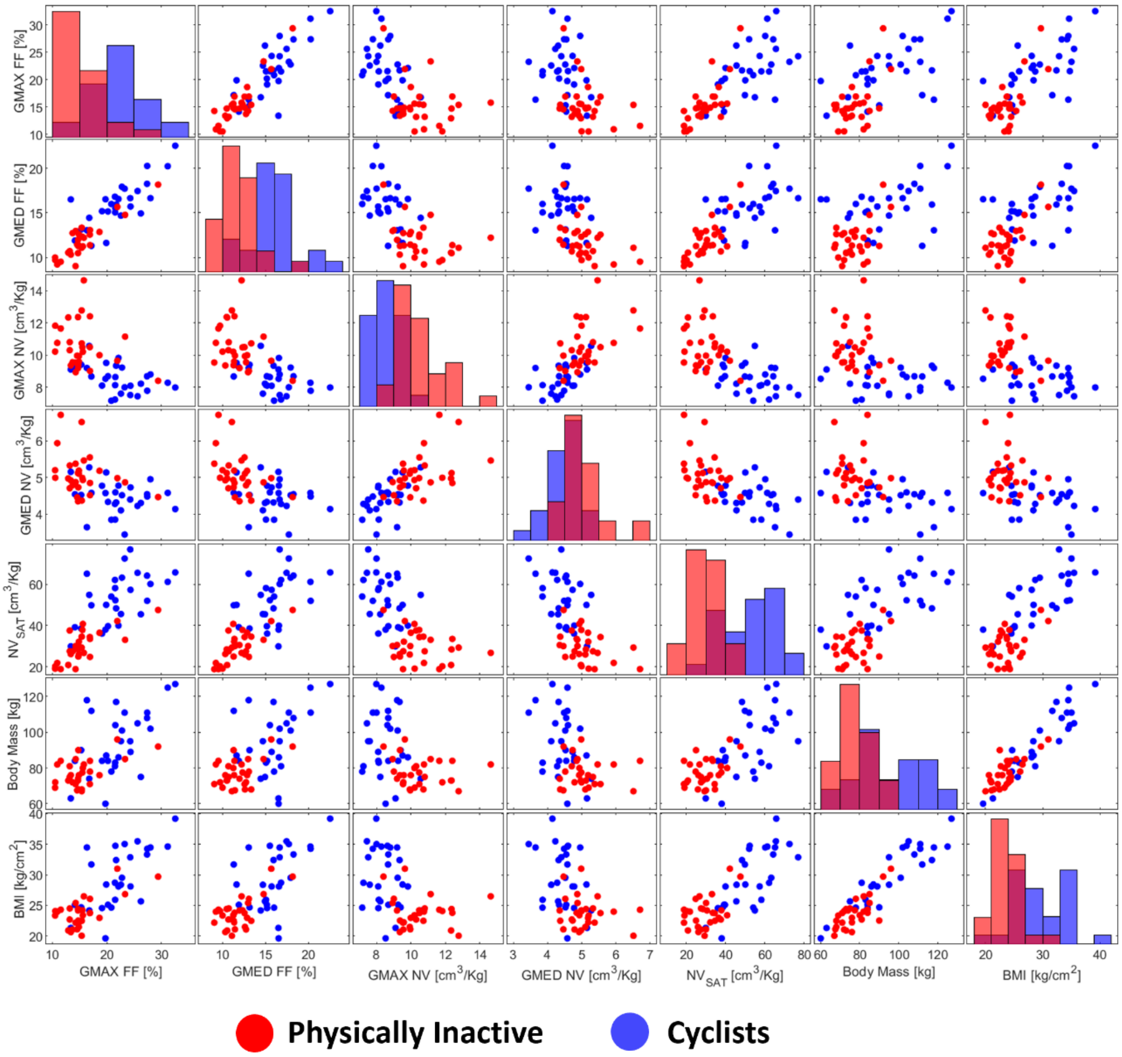


Figure S. 3. Exploratory data analysis of the main variables involved in muscle health for the physically inactive and cyclists groups.
